# Introduction: Disease Reservoirs: From Colonial Medicine to One Health

**DOI:** 10.1080/01459740.2023.2214950

**Published:** 2023-07-31

**Authors:** Matheus Alves Duarte da Silva, Oliver French, Frédéric Keck, Jules Skotnes-Brown

**Affiliations:** a Department of Social Anthropology, University of St Andrews, St Andrews, UK; b Laboratory of Social Anthropology, Centre National de la Recherche Scientifique (CNRS), Paris, France

**Keywords:** Colonial medicine, emerging diseases, global health, One Health, reservoirs, zoonosis

## Abstract

The introduction of the special issue “Disease Reservoirs: Anthropological and Historical Approaches” sets out the origins and trajectories of disease reservoir frameworks. First, it charts the emergence and elaborations of the reservoirs concept within and across early 20th-century colonial contexts, emphasising its configuration within imperial projects that sought to identify, map and control spaces of contagion among humans, animals, and pathogens. Following this, it traces the position the reservoir framework assumed within post-colonial practices and imaginaries of global health, with particular reference to the emerging infectious disease paradigm. The introduction shows that, in contemporary usages, while the concept continues to frame animals, humans and their bodies as containers of previously identified pathogens, it also emphasises the imperative of anticipating as-of-yet unknown diseases, harboured in the bodies of certain animals, through networks and techniques of surveillance. Consequently, the introduction argues that the notion of disease reservoirs remains intimately intertwined with concerns over the classification, organization, and management of peoples, pathogens, animals, and space. Finally, the introduction outlines the seven papers that form this special issue, stressing how they dialogue, complement, and challenge previous historical and anthropological approaches to disease reservoirs, with an eye to opening up new avenues for cross-disciplinary exploration.

The notion of a “disease reservoir” has become a dominant concept in epidemiology and microbiology in the past century to conceive how pathogens circulate among animals, humans, or insects undetected, before jumping to a different animal population or to humans, causing outbreaks. While it retains the modern idea that a disease is linked to a specific pathogen, often transmitted in a linear chain of contagion, it simultaneously points to notions that a disease finds its origins in a complex set of relations where pathogens emerge, in a way comparable to older European medical conceptions of miasmatic milieux. The theoretical hypothesis that a disease is left unexplained if its animal reservoir is not found often leads to interventions aimed at monitoring, controlling, and sometimes eradicating the reservoir. In sum, the notion of a disease reservoir sits between the quest for origins, which often misleadingly attributes blame for an epidemic, and the modern concern for the protection of infrastructure, which more pragmatically prepares for future epidemics.

This idea that particular animals, but also particular human populations, and environments harbor or distribute diseases is complex and has profoundly shaped an array of multispecies relationships in the twentieth century and beyond. In this special issue of *Medical Anthropology*, launched after a workshop organized at the University of St Andrews on 26–28 May, 2021, we have gathered a series of ethnographic and historical case studies on disease reservoirs, which think critically about the contemporaneous uses of the concept, while reflecting on its emergence within and between heterogeneous contexts. Indeed, our contention is that anthropology should rely on the archival methods of history to “de-naturalize” (Packard and Brown 1997) the idea of a “disease reservoir” and analyze the instability of this concept in order to tame the unpredictability of zoonotic pathogens (Lynteris 2013).

In this introduction, we firstly chart the historical emergence of this framework. We show that in its origin, the notion of disease reservoir was linked to medical practices in modern colonial empires that sought to map and control spaces of contagion among humans, animals, and pathogens, and to understand the presence and the recurrence of outbreaks of certain diseases in some regions of their colonies. The idea of reservoir was applied to humans and animals alike, living in geographically contained localities, and to pathogens previously detected by colonial doctors, such as malaria in Algeria and trypanosomiasis in South Africa. From the 1920s onwards, without necessarily losing its linkages with human populations, the concept gained new meanings, being used to frame moving, and even “global,” animals such as rats and migratory birds. In this alternative conceptualization, the reservoir appeared associated with the animal body rather than a particular environment or human populations. We argue, therefore, that in its origins, the concept of reservoir has woven together human populations, animals, and environments, through attributing blame to elements of these assemblages for generating and conserving pathogens.

After examining this genealogy, the introduction reflects, secondly, on how the concept of reservoir has evolved within post-colonial practices of global health and the paradigm of emerging infectious diseases. In its contemporary usages, while the concept remains attached to animals, humans and their bodies carrying already identified pathogens, it also suggests that certain animals may harbor unknown diseases which may be anticipated by new techniques of surveillance. As our historical and ethnographic examples show, in seeking and identifying certain landscapes, peoples and animals as “reservoirs,” pathological associations, and racial imaginaries which were forged between imperial, medical, and moral concerns are powerfully reinscribed. We posit that the notion of disease reservoirs is thus intimately intertwined with concerns over the classification, organization, and management of peoples, pathogens, animals, and space.

In sum, the idea of disease reservoirs and its concrete effects indicate an ontological separation between nature and culture, a complexity that, we argue, can better be understood by a combination of historical and ethnographical accounts. As the papers gathered here show, in most of the cases, nature was/is understood as the place where disease was/is conserved among animals (Santos’ and McKay’s contributions to this volume challenge this assumption, nonetheless), and humans appear/ed as the victim of diseases. Nonetheless, the ontologies at stake were plastic and the papers show how some animals were conceived as more pathological than others, whereas some human populations became more associated with nature and therefore with diseases.

## The emergence of reservoirs

The word reservoir first appeared in France in 1510 and referred originally to a receptacle that contained a liquid. In the following centuries, the term evolved in line with its use in engineering, encompassing storage places for grains and spaces, real or metaphorical, that contained a given substance or entity.^1^ These two meanings are linked in the current definition given by the *Oxford English Dictionary*: “a natural or artificial lake where water is stored before it is taken by pipes to houses.”^2^ Colonial debates on the occupation of the land reveal that water tanks for securing inhabitants from droughts and floods predated the arrival of Western colonizers and were objects of discussions on how to improve infrastructures of water storage (Sivasundaram 2013:238–243). In medicine, the notion of a disease reservoir emerged at the end of the nineteenth century, gaining traction in the first decades of the next century to ascribe origins to diseases that affect human populations in places where they are contained, in a form of what we might call “natural storage.” According to Louis Pasteur, variation of virulence explained the pathogenicity of microbes following rules of immunity: frequent encounters with a microbe produced a memory (antibodies), which protected the organism against future encounters (Moulin 1991). The transmission of microbes from animals to humans, in particular, produced such variations of virulence, as showed by the use of vaccines from cows or the use of experimental animals, such as rabbits, dogs, or guinea pigs. Nonetheless, the idea of disease reservoir did not have much traction on metropolitan France at the turn of the twentieth century. Rather, the assumption that animal and human populations harbor pathogens to which they seemed immune came from colonial settings, especially in Africa, being deeply connected with practices of separation between human populations, and between humans and animals. Human and animal relations were strongly racialized within this period – with colonists framing indigenous people as closer to nature and thus more likely to be in contact with pathogenic animals or substances (Newell 2020; Saha 2021). This led to calls from medical professionals for the removal of racialized groups from city centers, and their segregation from White populations on medical grounds (Swanson 1977).

In the French Empire, the brothers Edmond and Étienne Sergent, working on malaria in Algeria, were central to the construction of the concept of reservoir. In one of its first mentions in the *Annales de l’Institut Pasteur*, in 1905, the Sergents wrote that, in Algeria, “the virus reservoir [of malaria] was constituted by 1) past infected Europeans and 2) by the indigenous, especially indigenous children, very often infected without morbid manifestations” (Sergent and Sergent 1905:129). The term reservoir appeared here not alone but inside a phrase – “réservoir du virus” or “réservoir de virus” – a pattern that remained in use in Sergent’s paper (Sergent and Sergent 1910) and other authors’ research (Tanon 1922). Virus was still an elusive concept in the 1900s, nebulously referring to a poison that induced illness, a bacterium, or a micro-organism that cannot be cultivated, nor seen through the microscopic, and whose effects were only perceived by its passage through organisms, such as smallpox (Duclaux 1898:32–37). The Sergent brothers knew that malaria was caused by the plasmodium – a protozoon described by Alphonse Laveran and whose presence they had identified in the blood of Europeans and Africans (Sergent and Sergent 1905:130). Henceforth, the “réservoir de virus” of malaria, in this case humans in colonized Algeria, contained the microbiological source of malarial infection in an apparent latent stage, which could be a source of new outbreaks among French colonists with no previous immunity if the “reservoir” was bitten by the Anopheles mosquito. In this early and influential configuration, the “malaria reservoir” was not so much a fixed subject but a complex web of relations that connected different human populations, mosquitos, and the plasmodium.

A second direction of the research on disease reservoirs concerned the role of animals in “harboring” a “virus,” that could spread to other species, a question that was being developed in parallel and with longstanding results in the British Empire. One of the earliest adoptions of the idea of an animal reservoir of disease in British imperial tropical medicine came from a small and isolated hill station in the Lebombo Mountain Range in Zululand, South Africa. In 1891, Zulu cattle-keepers complained to the colonial state that legally protected big game animals were spreading *unakane*, a fatal cattle disease, to their livestock (Brooks 2001:176–81). Both colonial officials and African farmers were divided on whether the disease (anglicized as *nagana*) was caused by the bite of the notorious tsetse fly or whether it was transmitted by large wild animals. Although the blood-sucking tsetse fly had long been indicted by British big game hunters and explorers as a pestilent insect, the idea that African “big game” were sources of a livestock disease was an unprecedented and controversial claim. British ruling classes commonly regarded these animals as majestic and beautiful creatures for the wealthy to hunt, mount, and often consume (Carruthers 1995; MacKenzie 1988). Distrustful of Zulu healers and herders, the Colony of Natal thus commissioned Surgeon Major David Bruce to investigate in 1894. Bruce, working with his wife Mary Bruce and a team of Zulu fieldwork assistants, hunters, laborers, and cattle herders spent two years investigating the problem and published two reports on their findings which, despite dismissing Zulu ideas as “not very trustworthy,” essentially corroborated the Zulu complaints – big game were carriers of the disease which was caused by a parasitic microorganism (later named *Trypanosoma brucei*) and circulated between game and livestock via the bite of the tsetse fly (Bruce 1896).

In his initial report, Bruce did not use the word reservoir to refer to big game. Instead, he drew upon a different water metaphor: the harbor. In 1896, he wrote that “big game harbor the parasite which causes the disease for a longer or shorter time with little or no disturbance to health” (Bruce 1896:19). In 1905, in concurrence with Edmond and Étienne Sergent, Bruce began using the term “reservoir,” referring to big game explicitly as a “reservoir of the disease” at a joint conference of the British and South African Associations for the Advancement of Science in Cape Town (Bruce 1905:298; for context see Dubow 2000:66–99). This proved an extremely controversial point as it was a period in which a British imperial conservation movement, spurred by the Society for the Protection of the Wild Fauna of the Empire (SPWFE), was advocating the protection of such animals from their perilous decline brought about by imperial overhunting (Carruthers 1995; MacKenzie 1988). Essentially, Bruce was framing imperial game reserves as vast breeding grounds for tsetse flies and reservoirs of nagana.

What ensued was a feverous debate that elicited hundreds of responses in scientific journals, conservation publicity, newspapers, and popular periodicals such as *The Spectator*, *The Field*, and *Country Life* over whether “big game” could constitute a reservoir of disease and under what conditions (for analysis pertaining to tsetse flies, see Mavhunga (2006). This long-standing debate between bacteriologists, zoologists, ecologists, farmers, and hunters continued well into the 1930s (Brooks 2001; Brown 2008). The wide-reaching nature of this discussion almost certainly played a role in circulating and familiarizing English periodical-readers around the world with the concept of a reservoir of disease. However, despite its circulation within these print communities, the origins of this theory – within Zulu etiologies of disease and practices of colonial game protection in nature reserves – were entirely lost. The formulation of the concept of reservoir in British Imperial Tropical Medicine thus owes part of its genesis to extractive research which repackaged indigenous southern African ideas within Western medical vocabularies and stripped them of their origins.

The identification of nature reserves as reservoirs was imbricated in programs of colonial governance, drawing attention to the ways the concept may work to justify the appropriation of land and livelihoods. Indeed, as a number of works in the issue will elucidate, the establishment of disease reservoirs has been intimately intertwined with epistemological violence and prejudicial logics of blame. The explosion of research into rats during the Third Plague Pandemic (1894–1959), an important catalyst in the emergence of the concept, clearly illustrates this connection. Hitherto seen as unrelated to disease, varied accounts of “unusual” rat mortality increasingly figured in narratives of plague, frequently related as precursors to human outbreaks. Despite the publication of Paul-Louis Simond’s famous rat-flea transmission experiment in 1898 (now shown to have been botched), the epidemiological status of rats was a matter of debate in those first years, with some experts considering them as fellow victims of a broader environmental infection, while others blamed them for spreading plague by their excrements or by coming in contact with food and cloth (Hankin 1898; for Simond’s experiment see Lynteris 2022). Rather than damp reservoirs of disease maintenance, rats and fleas were often envisaged as animals which spread plague like “a wildfire” that needed to be stamped out (Brunton 1907:37). Interestingly, the term reservoir was rarely applied directly to rats during the first decades of the third plague pandemic, even as vast networks of scientists, colonial officers, capitalist entrepreneurs, and fumigating machines were mobilized around their apprehension, control, and eradication (Engelmann and Lynteris 2020). Nonetheless, the relating of rats and plague catalyzed the proliferation of international and imperial scientific and commercial networks, occasioning a series of epidemiological notions and practices which became fundamental to later configurations of the “disease reservoir.”

In British India, where the pandemic claimed 12 million lives and formed an important focus for plague research, the location of plague within the bodies of rats continued to embed a constellation of established sanitary concerns, such as dampness, filth, stale air, darkness, and grain. Moreover, whilst the rat, and to a more limited extent its flea, *Xenopsylla cheopis*, were increasingly popularized and reviled as the “agents” in the maintenance and dissemination of plague, explanations for their pathological presence reinscribed racial and classed stigmas, an approach Prakash Kidambi (2004) has termed “contingent contagionism.” Thus, the identification of rats as primary agents of plague elaborated a prejudicial matrix of “blame,” through which pathological imaginaries of racial degeneracy, allegedly primitive “native” lifeways and the reproduction of poverty were bound together and reified (Evans 2018).

The observation that rats could carry plague bacilli in an attenuated form circulated to France, where it gained a new dimension after plague broke out in Paris in 1920. To face this outbreak, the Préfecture de Police created a laboratory responsible for examining rats caught in the French capital in search of the plague bacillus. Among 5000 rats captured between 1921–22, Louis Tanon, a medical doctor in charge of autopsying rats, found that 31 presented the plague bacillus in their bodies; however, surprisingly, they were healthy and did not present any plague lesions in their body organs. Therefore, Tanon concluded that “the rat appears as a virus reservoir” of plague, because it “can conserve, throughout the years, the [plague] bacillus in its body organs, transmitting it to next generations in a less virulent form, until when, on the influence of second causes, [the bacillus] retrieves its activity slowly, causes an important outbreak among its hosts, and then attack the man via the flea” (Tanon 1922:250). Tanon’s “réservoir de virus” seems thus slightly different when compared with the Sergents. Indeed, while the latter considered that a prior and even severe sick state was necessary to constitute a reservoir, for Tanon, a virus reservoir was instead comprised of “organisms that, although hosting a pathogenic microbe, are refractory to its action in habitual conditions,” being therefore closer to Bruce’s approach (Tanon 1922:254). Perhaps, this influence was a result of the circulation of knowledge between British, French, and German scientists working on trypanosomiasis in East Africa (Ehlers 2019). However, whereas both Bruce and the Sergents feared specific populations harboring pathogens in restricted (if occasionally transnational) spaces, Tanon’s approach seemed to point to the risk of plague reservoirs emerging around the globe, given the deeply rooted belief that rats were good migrators, and especially partial to inhabiting ships (Skotnes-Brown 2023).

In a parallel and independent movement, Ernest Conseil and Charles Nicolle proved the role of lice in the natural transmission of the typhus by successfully inoculating monkeys with lice in the laboratory, and recommended cleaning houses and clothes and fumigating hospitals with sulfur. They wrote in 1911: “These prophylactic measures should be particularly directed against persistent reservoirs, even before the epidemic spreads. Knowing these reservoirs and their usual locations in winter will facilitate the application of rigorous measures to eliminate them.” (cited in Pelis 2007: 73). Nicolle had unsuccessfully searched for the origins of leprosy in fish after he had observed that it affected people who lived close to the sea (Huet 1998:70). In 1919, Nicolle and Charles Lebailly coined the term “unapparent diseases” after successfully transmitting typhus to guinea pigs, which carried the disease without symptoms, and warned Europeans of the risks of disease transmission after the movements of population during the war (Nicolle and Lebailly 1919). Nicolle was probably the first to imagine the mechanisms of microbial mutations explaining the life cycle of infection, and to prophesize that there would be new infectious diseases (Nicolle 1930).

During the 1920s and 1930s, the “reservoir” concept underwent important development outside the borders of the British and French Empires and within the United States and the Soviet Union. Theobald Smith, whilst acting as the Director for the Department of Animal Pathology at the Rockefeller Institute, extended the concept to domestic animals. He wrote in 1928:

The animals which contribute to human disease may be divided into useful and noxious species. From a scientific viewpoint the distinction is of no value. It becomes, however, very significant, when we endeavor to suppress the diseases. We may make continuous, relentless warfare on rats and mosquitoes, but the problem becomes more complex when we deal, for example, with cows as reservoirs of human diseases. Medical literature abounds to the possibilities of harm lurking in animal diseases, and in nearly every great epidemic of the past, animal diseases have been reported as precursors. (Smith 1928:477)

This work elaborated an important and lasting notion: that transmissions of pathogens from animals to humans are rare and the conditions under which they occur remain mysterious. Preempting later concerns with “anticipating” outbreaks, Smith asserted the urgency of investigating the mechanisms of parasite change to see diseases before they appear: “the ancestry might be directly before us, in our midst, in fact some animal disease, but we may fail to see it because of the irreversible process that has brought the change about” (Smith 1928:496).

The Swiss-born veterinarian Karl Friedrich Meyer, who trained at Harvard University with Theobald Smith, published in 1931 an important article entitled “‘The Animal Kingdom – A Reservoir of Disease’” (Meyer 1931). Meyer had traced the transmission of botulism by spores in the soil, of plague by squirrels, of equine encephalitis by mosquitoes and of psittacosis by parrots (Honigsbaum 2016). He developed a correspondence with the Australian microbiologist Frank Macfarlane Burnet, who had identified the chlamydia-causing psittacosis, and was to become one of the main theoreticians of the ecology of infectious diseases, with a view of animal reservoirs as ecosystems that should be regulated and protected that differed from Meyer’s (Anderson 2004). While Burnet drew on Darwinian ideas of competition between predator and prey reaching an equilibrium, and the much older idea of a “balance of nature,” Meyer, following Smith, talked about mutually beneficial relations between parasite and host, producing infections he called “latent” or “inapparent.” But like Burnet and following the ideas of the ecologist Charles Elton, Meyer conceived infections as regulators bringing ecosystems back to an equilibrium. He wrote that those “who by necessity were forced to interpret the dangers of infection which emanate from the vast reservoir in the animal kingdom fully acknowledged the guiding hand in the ecological concept of the epidemics produced by population regulators – the microbial or virus parasites” (Meyer 1941:348).

An alternative formulation of the animal reservoir appeared in parallel with the concept of natural nidality in the Soviet Union, a concept that became embroiled in the application of intensive programs of landscape management. It was developed in the 1920s and 1930s and spread to the United States through the publication in 1966 of the translation of Evgenii Pavlovsky’s work, *Natural Nidality of Transmissible Diseases*. Pavlovsky taught zoology at the University of St. Petersburg (Leningrad) from where he organized expeditions to Central Asia, which led him to be elected president of the Geographical Society of the Academy of Sciences and to receive the title of Hero of Socialist Labour in 1952. The theory of natural nidality allowed him to explain the conservation of the plague bacillus among marmots and other rodents, as well as its persistence in burrows and caves. Pavlovsky spoke of parasitocenosis to describe the way in which an organism, conceived as a habitat for the microbe, became part of a secondary environment, composed of the relationships between animals, plants, and soil, where an invisible chain of transmission takes place. This dynamic community of organisms that formed a nest (*ochag*) for microbes constituted for Pavlovsky an unstable equilibrium requiring constant monitoring of environments. The politics of agricultural development led the Soviet government to apply this theory by sanitizing the landscape and liquidating the components of the outbreak, including removing human populations that might contribute to the transmission of the disease. The theory of natural nidality thus led to an interventionist approach on land use for agricultural development, ignoring local conceptions of interactions between humans, animals, and microbes (Jones and Amramina 2018).

In sum, it was during the first half of the twentieth century that the notion of “disease reservoir” emerged as a nebulous idea which variably framed differing understandings and approaches to pathology. Overwhelmingly, it was in animals that the sources of human infection were sought, identified, and acted against. Yet in seeking the “reservoirs” for disease, diffuse forces and agencies, from landscapes, water, air, and human populations were configured as variables that could explain the transmission and maintenance of pathogens. Colonial representations of disease reservoirs conceived nature as a set of phenomena endowed with its own regulation and yet as a space that needed intervention to control its disruptions. This understanding was not limited to what we could call today animal ecosystems but was frequently extended to “native villagers” as targets for colonial intervention. In that sense, the concept of reservoir often blurred the boundaries between humans and animals and between nature and culture, framing some human populations as closer to animals than others, and therefore more responsible for maintaining pathogens. How, then, did the end of formal European empires and the agency this provided to former colonized populations transform the concept of the disease reservoir? And how did this process configure the notion of emerging infectious disease as we know it in contemporary global health?

## Reservoirs and emerging infectious diseases

The notion of “disease reservoir” played an important role in the framing of global health as an attempt to anticipate the appearance and spread of uncontrollable pathogens in the 1970s. Central to this “pandemic imaginary” was the perceived capacity of new pathogens to emerge on any point of the planet, spreading rapidly to human populations across the globe through ever denser networks of connections in a way that challenged the rationalities of public health based on calculation of risk and prevention (Lakoff 2017; Lynteris 2021). More often than not, however, the sites and spaces in which these new pathogens were prospected and sought out continued to be inflected by patterns of prejudice and stigma instituted by colonial configurations of reservoirs. The idea of emerging infectious diseases, crafted between 1989 and 1995, sustained the framing of Africa, Asia, and South America as critical regions for the generation, maintenance, and dissemination of novel pathological threats (Silva and Skotnes-Brown 2023). A now familiar pattern describes the emergence of a new disease, such as Ebola in 1976, as the starting point for a race to identify and isolate the virus that caused it and to manufacture drugs and vaccines to treat and immunize against it (King 2002). While standardization techniques were on the side of pharmaceutical companies targeting new pathogens, they were also harnessed by microbiologists to trace the genetic sequences of microbes. Robert Webster, who found antibodies for influenza among wild birds in Australia in the 1960s, launched a global quest to map the genetic mutations of influenza causing changes in its HA and NA molecules and in their capacity to infect humans (Webster 1992). He described “the sheer magnitude of the avian reservoir” as a “gold mine” for collecting and storing viruses (Webster 2018:45).

This project to map and treat emerging pathogens, which defines global health as a network connecting labs and clinics, is linked not only to novel technologies of genetic sequencing or the “molecularization of life” (Braun 2007) but also to the redefinition of diseases as targets of biosecurity interventions (Lakoff and Collier 2008). “Target populations” are defined as populations smaller than the critical size for the survival of the pathogen, where it can temporarily “jump” before returning to its “natural” reservoir. A connected but somewhat distinct notion is the “epidemiological dead end:” a species into which the pathogen “arrives” but from which it fails to transmit to another. In zoonotic diagrams (Lynteris 2017), animals are invariably configured as the sites of these random mutations, humans figuring as the ultimate targets of emergence (Haydon 2002). The concept of target plays on both sides: in the description of routes of transmission from the reservoir and in the intervention on the reservoir to contain the infection. While global health is concerned by the life and death of an emerging infectious disease, the notion of disease reservoirs was reshaped to understand the survival of the disease after its end, in a form of undead existence: “an ecological system in which the infectious agent survives indefinitely” (Ashford 1997). As global health implemented techniques of imagination to represent what cannot be calculated because of the immense range of microbial mutations (Lakoff 2017), the emergence of a zoonotic pathogen is conceived as an event with catastrophic consequences, as a “spillover” from the reservoir that causes an “outbreak” in the target population, harnessing the powers of fiction and simulation such as novels and films (Wald 2008). Zoonotic infection is thus configured as a rupture in the “natural” systems of pathogen maintenance through the impact of the human species on its environment through industrial breeding or deforestation. The idea of storage ingrained in the reservoir concept, intensified by accelerations in ecological changes, is contrasted with the notion of stockpiling in the techniques of global health, as a set of rules of priority in the management of therapeutic tools targeting an emerging pathogen, such as vaccines, antivirals, and masks. Virologists thus store viruses in viral banks in the attempt to simulate their natural mutations and anticipate their zoonotic outbreaks (Keck 2020).

This crucial distinction between the reservoir and the target thus leads to two contrasting modes of intervention. On one side, measures are taken to swiftly cut the connections between humans and disease reservoirs through the destruction of the latter, encapsulated by methods such as the wholesale culling of poultry to control the spread of avian influenza. These spectacular measures to “eradicate” the reservoir are accompanied by more mundane measures of biosecurity to instantiate distances in spaces where humans risk being transfigured into siphons for “animal” diseases. This mode consists of the identification, regulation, and surveillance of spaces positioned as dangerous interfaces between human and suspected “reservoirs,” for example, poultry farms and wetmarkets where there is a risk of infection by highly pathogenic avian influenza such as H5N1 (Cardona et al. 2009; Fearnley 2020; Porter 2019). Anthropologists have repeatedly critiqued how racially charged imaginaries frequently animate the identification of these spaces and practices of animal-human interaction. Bush-meat hunting, for example, a term ensnarled in colonial racist classificatory schemas, is frequently framed as an unregulated practice, inevitably undertaken in the global south, which elides the safe distance between human and wild animal populations, consisting of both “known” and potential new reservoirs (Thys 2019). Deployed as a vague evocation of a mode of contact inviting a “spill-over” event or a “hotspot” of declining biodiversity correlated with high risk of viral emergence (Schmidt and Ostfeld 2001), the historically embedded patterning and material interactions between humans and animals underpinning pathogenic spread are obfuscated (Brown and Kelly 2014; Narat 2017). Imbricated in an imaginary of the “wild” as the true source of disease, the preoccupation with the hunting trope likewise imbues “bushmeat” communities with an identity as being closer to and unwitting conveyors of this pathogenic realm. This said, it can also be reframed as a form of collective vigilance on pathologies detected at the borders between species through early-warning signals and sentinel devices (Keck 2020).

Similarly, in cases of human-transmitted diseases, attempts to contain reservoirs within particular regions have involved racially and xenophobically charged biosecurity measures. During the first two years of the COVID-19 pandemic, wealthy countries swiftly implemented travel restrictions to and from regions in which new and dangerous variants were identified for the first time while simultaneously hoarding vaccines and critical therapeutics. The effect was that countries such as South Africa, Brazil, India, and many others were stigmatized as reservoirs of dangerous mutations of Covid-19, blamed for perpetuating the pandemic, and isolated rather than supported. An important counterpoint was the UK, the place where the first variant of concern was identified, which led to occasional bans of air travel to the country and disruption of non-essential international travel. Nonetheless, travel bans and restrictions imposed by other countries upon the UK were enforced for a shorter time when compared with those applied to other places where variants were originally identified. This suggests that geopolitical affinities continue to overcome sanitary concerns in the contemporary logic of early warning signals.

In contemporary global health, a disease reservoir is conceived not only as a space to keep clean or separate but also as an ecosystem that should be preserved. As we have seen in the South African genealogy of the concept, the study of reservoir species is animated by an interest in conservation of wildlife, beyond the colonial space of the natural reserve. The concept “One World, One Health” was promoted by the Wildlife Conservation Society in 2005 to coordinate the efforts of international organizations such as the WHO, the FAO, and the OIE to monitor and control the spread of avian influenza from Asia to the rest of the world, by paying attention to the ecosystems in which wild and domestic birds can be kept healthy (Hinchliffe 2015). Yet, historians have showed that the “One medicine” idea was promoted long before by veterinarians, for instance, by Calvin Schwabe in his book *Veterinary Medicine and Human Health* in 1964 (Woods 2023), and that Julian Huxley had stressed the necessity to monitor zoonoses in wildlife when he founded the International Union for Conservation of Nature (IUCN) in 1948 (Lainé and Morand 2020). Thus, bats have been studied in southeast Asia as reservoirs for coronaviruses or Henipah viruses, because they live in colonies where they exchange viral strains and have developed immune systems for flight allowing them to carry viruses asymptomatically (Wang and Cowled 2015). Bats are often protected species handled with care, but some humans kill them or chase them to protect themselves against infection, which makes them particularly problematic disease reservoirs mixing different epistemological and ontological perceptions of relations between humans and animals (see Roth’s contribution to this volume).

The orientation of global health initiatives toward the anticipation of disease emergence through the surveillance of non-human reservoirs has recently been complicated by the phenomenon of “reverse zoonosis.” Far from the description of humans as inevitable targets or dead ends of epidemic transmission, the trajectories of microbial flows among certain human bodies and socialities are represented as the receptacles for the maintenance of diseases which now emanate toward animals. Thus, pig workers in Midwest industrial farms, when following biosecurity measures, must imagine themselves as reservoirs for Porcine Epidemic Diarrhea virus which killed 10% of pigs in the United States (Blanchette 2015). Or in the current outbreak of highly pathogenic H5N1 avian influenza in South America, domestic poultry has become the reservoir for deadly mutations infecting wild migratory birds (Bodewes and Thijs 2018). Rather than an isolated event, these pathogen transmissions through what is called zoonotic “spill back” raise questions on the co-evolutionary entanglements between species over greater temporal scales. In the face of continual “zoo-anthropic” transfers, locating the incipient reservoir is a fraught process. In his work on elephant conservation in Laos, for example, Nicolas Lainé (2018) highlights the difficulty public health officials faced in addressing suspected transmissions of tuberculosis between mahouts, tourists, and the elephants themselves. While some zookeepers pushed for “culling” the elephants to avoid transmission to humans, others stressed that they should be kept separate from tourists, or else simply considered that the disease was not a profound concern. Similarly, the transmission of COVID-19 to minks and deer from the human population has been targeted by different politics of biosecurity – deer are hunted and cannot be culled like industrial minks – but will most likely lead to the maintenance of COVID-19 as an endemic disease (Haider 2020).

Understanding how certain species and populations shift from being conceived as reservoirs to being conceived as targets is a powerful motivation for inquiries on the management of emerging infectious diseases. Even if the definition of disease reservoirs is inscribed in logics of blame and politics of villainization, each with their own intricate histories, it seems impossible to predict how the framing of a population or a species as a disease reservoir will lead to a particular mode of intervention. For instance, the institution of badgers as reservoirs for TB in Britain during the 1970s mobilized an array of responses, from cattle farmers, who largely argued for culls, to animal activists, who protested such measures. Over the decades, governmental interventions fluctuated drastically, forming along with the shifting political strategies and priorities of various governments during a politically tumultuous era. Significantly, throughout these oscillating approaches, and indeed to this day, the question whether the badger constituted a “true reservoir” or a “fellow victim” of TB was never fully established even within the departments responsible for their control (Cassidy 2019; Enticott 2001).

In sum, disease reservoirs associate a group or a species with space and values which can be negative or positive depending on the social construction of the disease. The diversity of contributions in this special issue seeks to reveal the complexity of the history and anthropology of disease reservoirs. The special issue begins with Matheus Alves Duarte da Silva’s reconstruction of the institutional, scientific, and imperial backgrounds of the invention of the concept of sylvatic plague in the 1920s and 30s and its global circulation in the 1940s and 1950s. The history and the meanings attached to the concept are often ignored by historians and medical anthropologists. Nevertheless, the concept not only became central in the twentieth century to think about and act upon plague reservoirs around the globe but challenged the microbiological paradigm by ascribing the identity of a disease not to its pathogen but to its reservoir and where it inhabits. By following its creator, the Portuguese doctor Ricardo Jorge, Silva argues that Jorge invented a general space of plague latency, described as desert-like environments, where wild rodents maintained the sylvatic plague independently from humans and “domestic” rats. The article discusses the meanings of the word “sylvatic,” arguing that it inherited from a Portuguese imaginary about the Amazon as a wild and empty space, and examines its ambiguities, mainly that of sylvatic being used to describe plague reservoirs in steppes and deserts. The article retraces the circulation of the idea to the Americas, showing how it was incorporated by Karl Meyer in California and specially by plague scholars working in Brazil, where sylvatic plague passed to mean in the 1940s a feared “jungle plague.” Despite these new iterations, Silva argues that the idea of sylvatic plague remained attached to spaces imagined as empty of humans and of domestic rats, and because of that, the idea of sylvatic plague at times reenforced, but also unsettled the ontological partition between nature and culture.

Both complementing and contrasting with Silva’s case study, Jack Greatrex’s analysis of colonial conceptions of plantations, rodents, *lalang* grass, and “jungle” in the 1920s–40s Federated Malay states shows how colonial plantations were constructed as reservoirs of scrub typhus. Demonstrating the influence of this conception on both the physical and intellectual landscape of Malaya, Greatrex complicates historiographical conceptions of tropical environments being framed as sites of disease. Greatrex shows that rather than the underdeveloped wilderness as a site of epidemiological risk, colonial scientific work was fraught with anxieties about imperial transformations of environments creating disease reservoirs. More broadly, Greatrex demonstrates how attention to often neglected colonial sites such as Malaya can offer an alternative historical lineage for the emergence of disease ecology as a science. Colonial Malaya thus constitutes a key site in the development of this field, beyond the familiar story of Karl Meyer in California (Honigsbaum 2016).

Moving from history to anthropology, Bruno Silva Santos’ article invites the reader through an ethnography of Guarani’s perceptions of rats and zoonoses. Drawing on his fieldwork with the Guarani-Kaiowás on the Jaraguá Indigenous Land, a small indigenous reserve inside São Paulo, Brazil biggest city, Santos shows that the Guarani do not perceive rats, in general, as potential threats to their health but only those living in the city, given their contact with pollution and non-indigenous ways of living. By exploring the tensions between urban and wild spaces and between health and pollution from a Guarani perspective, Santos criticizes a common view among Brazilian White populations that frames Indigenous peoples, their reserves, and ways of living as source of filth and backwardness. Interestingly, this Guarani critique of non-indigenous way of living finds some echoes in the former romantic views of Brazilian elites, which in the nineteenth century presented often cynically Indigenous people as symbols of a pristine nature affected by urbanization. It is thus in this complex web of relations and meanings, concludes Santos, that the Guarani perception of the city of São Paulo as the ultimate reservoir of disease should be understood.

In a dialogue with Santos’ article on how animals are constructed as disease reservoirs, Emmanuelle Roth explores the work of bat samplers in Forest Guinea in the context of uncertainties on the zoonotic transmission of Ebola. She demonstrates that while ecologists show that the dynamics of viral circulation make bats good reservoirs for the disease, virologists have found among these animal antibodies against the virus but not the virus itself, which may suggest that they do not replicate the virus. On the other side, local communities display little fear of bats but say they have stopped consuming them as bushmeat. Bat samplers must then act as if bats were virus-carriers when they wear protective equipment while avoiding being separated from local communities. This ambiguity vis-à-vis the bat associated with the need to ascribe a disease reservoir for Ebola are described by Roth as a “fetish,” a mix of material and semiotic traits producing alterity in a space of doubt, prudence, and misunderstanding.

Freya Jephcott gives a different response to the same question: how is a disease reservoir constructed in a situation of uncertainty? Following an investigation team on an outbreak of monkey-borne simian herpes virus in Ghana, she accounts for the reasons why the disease was inscribed in the forest even though it affected children living in a city. Drawing on the anthropology of rumors, she shows that epidemiologists were attracted to the idea of a monkey-forest reservoir, while they knew that no transmission from monkeys was possible. The notion of disease reservoir is understood in this account as a salient representation avoiding different actors to continue the investigation, leaving aside other causes such as the toxicological approach of the disease.

Melding ethnographic and historical research, Christopher Kelty considers the dominant, globally distributed infrastructure of rodent control: the bait station. Exploring its principal formulation in the post-WWII work of Charles Elton and the Animal Control Bureau, Kelty demonstrates how bait-stations manifested a conceptualization of “reservoirs” that moved beyond the bodies of animals, to the population dynamics of the “pests.” Carefully attending to the materiality of the box itself, Kelty reveals how this strategy departed from aspirations for wholesale extermination, instead promising masterful control through a “semi-permanent network” of stations. Although formed upon granular “scientific” knowledge of the intended victims, bait-boxes, both then as now, proved incapable of species-specific slaughter, emerging themselves as “reservoirs” of “secondary poisoning” in other species. Kelty makes a significant contribution to the polarizing structure of current debates, arguing that rather than a novel “solution” to this phenomenon, “ecological thinking” is intrinsic to this vision of rodent “reservoir” management. Drawing on their fieldwork in the rodent control industry in California, Kelty braids together the normalization of this structure with patterns of settler colonialism, alongside the temporality of rodenticidal substances and the “future proofing” logics of contemporary pest control capitalism.

Shifting away from animals and toward the study and management of human reservoirs in urban environments, Richard McKay’s article closes the special issue by examining the construction of the “male homosexual” as a reservoir of sexually transmitted infections (STIs) in North America. Drawing upon metaphors and concepts derived from sanitary and tropical medicine, as well as earlier fixations on female sex workers as STI reservoirs, public health officials in the USA and Canada identified male homosexuals as a particular epidemiological problem in the years following WWII. Through a close examination of the multifarious rhetorical, visual, and metaphorical meanings of the reservoir, McKay shows how the concept itself enabled and justified the biopolitical control of particular localities, groups of marginalized people, and even changes to the built environment of Vancouver. On the other hand, the fixation with “reservoirs of infection” led to epidemiological oversights, in some cases facilitating the further transmission of STIs. The idea of a reservoir of infection, MacKay stresses, as a historically contingent cultural and medical concept, with obscure and shifting boundaries, should be used with caution.

In conclusion, this introduction has drawn upon historical and ethnographic examples to illustrate the malleability of the disease reservoir concept. Whilst emphasizing the diverse configurations and applications of the notion, this issue suggests several important threads running through the many lives of the concept. In particular, we emphasize that the identification and apprehension of disease reservoirs has been a powerful mode through which microbes, organisms, and environments became related through pathological associations. Emerging within colonial classificatory and bio-political frameworks, the concept, whilst never a coherent entity, was variably deployed to denote certain spaces, practices, and beings as dangerous. These identifications simultaneously induced anxieties regarding the future of colonial projects, whilst also animating a proliferation of imperial, commercial, political, and scientific networks under the aegis of counter-epidemic activity. These associations and imaginaries did not simply dissolve with the formal end of Empires but continue to inform and animate global health frameworks of disease management. With the growth of a One Health perspective on zoonotic pathogens, this special issue invites anthropologists, historians, and scholars in general to pay attention to the varied ways in which the notion of “disease reservoir” reveals tensions in relations between species at a global scale, thus opening interesting potentialities and dangerous dead-ends in thinking about planetary health.

## Notes

1. Centre National de Ressources Textuelles et Lexicales. “reservoir, subst. masc.” https://www.cnrtl.fr/definition/reservoir.

2. Oxford English Dictionary. “reservoir, n.” https://www.oed.com/view/Entry/163518.
